# The effects of birth rank (single or twin) and dam age on the lifetime productive performance of female dual purpose sheep (*Ovis aries*) offspring in New Zealand

**DOI:** 10.1371/journal.pone.0214021

**Published:** 2019-03-21

**Authors:** E. J. Pettigrew, R. E. Hickson, S. T. Morris, N. Lopez-Villalobos, S. J. Pain, P. R. Kenyon, H. T. Blair

**Affiliations:** School of Agriculture and Environment, Massey University, Palmerston North, New Zealand; INIA, SPAIN

## Abstract

Greater rates of genetic gain can be achieved by selecting animals born to younger parents. However, little is known about the lifetime performance of dual purpose ewes (*Ovis aries*) that are born to primiparous ewe lambs (8 to 9 months old at breeding). This experiment investigated the effect of being born from either a ewe lamb or mixed age dam as either a single or twin on the lifetime performance of ewe progeny. Lifetime performance was measured in terms of the life time live weights of the ewes, the weight and number of lambs born and weaned, the efficiency of production (kilograms of lamb weaned / predicted pasture intake (kgDM) of the ewes), and ewe survival. The study followed the lifetime production of 17 single and 41 twin female lambs born to mature ewes (M1 and M2, respectively), and 28 single and 29 twin lambs born to ewe lambs (L1 and L2, respectively). Over their lifetime L2 ewes were lighter (P<0.05) but had similar body condition scores to the other three ewe groups. There was no difference in average progeny weaning weight or total progeny litter weaning weights between groups. The M1 ewes had the greatest longevity (P<0.05) of the four groups. Even though L2 ewes were lighter than the other three groups, this was insufficient to increase their lifetime efficiency of production (kg lamb weaned/predicted pasture consumption), relative to the other groups. These results suggest farmers could select replacements born to ewe lambs without sacrificing animal production.

## Introduction

Currently, 30–43% of dual purpose breeding ewes (*Ovis aries*) in New Zealand are bred for the first time as ewe lambs, at 8–9 months of age [1–5]. Ewe lambs are lighter and have lower body condition scores than mature ewes [6]. Lambs born to ewe lambs are smaller and lighter at birth [1, 4, 6–8], weaning [4, 9, 10], and to 12-months of age [4, 9], and have lower survival rates [7, 10, 11], compared with lambs born to mature ewes. Twin-born lambs born to mature ewes are also smaller and lighter than single-born lambs to weaning [7]. The same relationship occurs in ewe lambs [6].

Progeny born to ewe lambs have difficulty achieving a suitable breeding live weight as a ewe lamb, compared to lambs born to mature ewes, due to slower early growth [7], in addition they are born a month after lambs born to mature ewes in New Zealand [12]. Lambs born to ewe lambs are not commonly selected as replacements in New Zealand [12], with light live weight at weaning being the primary reason [7, 13–15]. Puberty is attained at 40–60% of mature live weight [7], or 38–48 kg in Romney ewe lambs [15]. Ewe lambs must have attained this percentage of live weight to have any chance of being successfully bred, and being able to support pregnancy and lactation requirements [7].

Single lambs born to ewe lambs have been reported to be lighter than singleton lambs born to mature ewes from birth to 12 months of age, and occasionally until four years of age [4]. Despite this, they produced similar numbers and weights of lambs at birth and weaning, and had a similar production efficiency (kg lamb weaned/estimated maintenance MJME). Loureiro [9] showed that for the first year of life, lambs born as singles to mature ewes were heaviest from birth to weaning, with twin lambs born to mature ewes and single lambs born to ewe lambs being intermediate and not different from each other, and twin lambs born to ewe lambs were the lightest. There was no difference in pregnancy rate, and number and weight of lambs weaned, for ewes born as either singles or twins, to either mature ewes or ewe lambs for their first lambing at two years of age [16], and as singles born to either mature ewes or ewe lambs for their first two lambings as two- and three-year-olds [17]. There was no difference in milk production from singles born to mature ewes or ewe lambs for their first two lactations [17]. Dual purpose ewes in New Zealand have an average expected productive life of 4.3–5.0 years [18]. However, little is known about the lifetime productive performance of dual purpose ewes that were born to ewe lambs, in New Zealand. Therefore, this experiment aimed to investigate the effects of birth rank and dam age on the maternal performance of ewes over six lambings. Lifetime performance was measured as the life time live weights of the ewes, the weight and number of lambs born and weaned, the efficiency of production (kilograms of lamb weaned / predicted pasture intake (kgDM) of the ewes), and ewe survival.

## Materials and methods

The experiment was conducted at Massey University’s Riverside Farm (latitude 40°50ʹS, longitude 175°37′E) 11 km north of Masterton, and Keeble Farm (latitude 41°10ʹS, longitude 175°36′E) 5 km south of Palmerston North, New Zealand, with the approval of the Massey University Animal Ethics Committee (MUAEC12/21). The experiment ran from April 2009 until January 2017. Animals were managed at Riverside Farm from September 2009 (birth) to January 2010 (weaning), then moved to Keeble Farm for the remainder of the experiment.

### Experimental design

The study used Romney nulliparous ewe lambs (L; 8–9 months of age) and multiparous mature Romney ewes (M; 3–5 years of age) as the parents of the experimental ewes. They were naturally bred with Romney composite rams (at a ram to ewe ratio of 1:40) as one cohort for a 34-day mating interval in April 2009 [6, 9, 16]. The ewes were grazed under commercial New Zealand grazing conditions, with post-grazing covers at a minimum of 1000 kg DM/ha during breeding and gestation and of 1200 kg DM/ha during lactation [6]. At weaning all female lambs were selected from resulting progeny to create four groups based on dam age (mature ewe or ewe lamb; M and L, respectively) and birth rank (single or twin; 1 or 2, respectively) [9, 16]. No triplet-born ewe lambs were selected from either dam age group. At the initiation of the main experiment, the progeny groups included single progeny born to mature ewes (M1, n = 17), twin progeny born to mature ewes (M2, n = 41), single progeny born to ewe lambs (L1, n = 28), and twin progeny born to ewe lambs (L2, n = 29). The four groups were managed as one mob (n = 115), from selection onwards [9, 16], and were followed for the following eight years across six lambing periods.

Ewes were first bred in 2011, at 18 months of age [16]. Rebreeding subsequently occurred once yearly from 2011 to 2016, between the 24th of March and 24th of April for different years. The ewes were treated with progesterone using CIDRs (controlled internal drug release; Pfizer Inc., New York, NY 10017) to achieve synchronised oestrus prior to joining with rams each year.

### Measurements

At birth, the ewe progeny had their live weight, thoracic girth, and crown-rump length measured within 12 hours of birth. The midpoint of the lambing period was defined as day 1 (d1). Further live weights (LWT) were recorded at day 41, weaning (d99), and monthly until their first breeding (d552) [9]. In later years live weights were recorded pre-breeding, at pregnancy detection in mid-pregnancy, one week prior to lambing, and at weaning. Body condition scores (BCS; 1 = emaciated, 5 = obese; [19]) were measured twice prior to the ewes’ first breeding (d369 and d412), and at each subsequent live weight.

Pregnancy diagnosis via transabdominal ultrasonography to count the number of foetuses present occurred each year between 72 and 86 days after ram introduction. Ewes were checked twice daily during the lambing period, beginning seven days before the planned start of lambing, and the birth weight, crown-rump length, and girth of their progeny were measured. Lambs had additional live weights measured at day 40 of lactation and weaning (average age of 99 days). No culling of ewes occurred unless on welfare grounds. Ewe deaths were recorded.

### Data handling

Live weight of ewes during pregnancy were adjusted via Gompertz equation [20] to calculate a conceptus-free live weight, based on birth weight of the litter and lambing dates. A transformational regression was used to fit a spline polynomial (order 2) curve to the adjusted live weights of each individual ewe. Spline knots were placed at Day 0, Day 99, Day 188, Day 337, and Day 432 prior to first breeding, then at breeding, pre-lambing, and at weaning each year, until the end of the experiment. A daily live weight prediction was generated for each ewe from their weaning as a lamb, until their death, or the end of the experiment (Day 2623).

Progeny weights were added together to form a total litter weight per ewe per year for birth, day 40 of lactation, and weaning weights. Total litter weight of lambs at weaning divided by the weight of the ewe at breeding, for each year to determine a ratio of progeny weaning weight to ewe breeding weight. If the ewe was present at breeding, but did not wean a lamb, a litter weaning weight of zero was given.

Predicted daily live weights from the spline models were used to determine the daily nutritional requirements for each ewe for their maintenance and growth/loss [21]. Litter birth weights and birth dates were used to determine daily nutrient requirements for gestation [20]. Lactation requirements were modelled from Peart [22] based on week of lactation, and number of lambs reared. Lamb nutritional requirements were modelled based on birth date and weight, weaning date and weight, and average daily gain [21]. Equations for daily energy requirements are presented in S1 Appendix. Total lifetime number of lambs weaned was calculated by adding yearly number of lambs weaned for each ewe. Similarly, the total lifetime weaning weight of lambs was calculated by adding yearly weaning weight of lambs weaned for each ewe. A total estimated lifetime feed requirement was calculated for all ewes, along with their lifetime litter weaning weight, the values were utilised to generate an estimated feed efficiency value for each ewe.

As the ewes were only culled based on welfare grounds, hypothetical culling was imposed retrospectively on ewes, if a particular ewe was barren at pregnancy diagnosis, day 40 of lactation, or weaning, as per commercial farming conditions. This allowed survival analysis to be carried out for actual survival of the ewes, and the expected survival of the ewes if they were managed under commercial farming conditions. All ewes alive for actual survival or imposed-culling survival at the end of the experiment (Day 2623) were censored at that date for survival analysis.

### Statistical analysis

Statistical analysis was carried out using SAS version 9.4 software (SAS Institute, Cary, NC, USA). The aim of this study was to classify production consequences of retaining single or twin ewes born to either a ewe lamb or a mature ewe. Thus, in all models, even if the interaction between dam age and birth rank was non-significant (P>0.05), the two-way interaction remained in the model, to allow for testing of the experimental question. Ewe live weights and some production traits up to 2.5 years of age have been previously reported by Loureiro [16] and Loureiro [23].

Ewe weight at birth, 41 days of age, and weaning, crown rump length, thoracic girth, progeny litter birth weight, progeny weight of the litter at 40 days of age, progeny litter weaning weight, ratio of progeny weaning weight to ewe breeding weight, total lifetime progeny weaning weight, total lifetime pasture consumed, and efficiency were analysed using a mixed linear model. The model included the fixed effects of age of dam (mature ewe vs ewe lamb) and ewe birth rank (single vs twin), and their interaction. Date of birth was included as a covariate in the model for the analysis of ewe weight at birth, 41 days of age, and weaning, crown rump length and thoracic girth. The model to analyse progeny litter weights at birth, 40 days of lactation, and weaning, and the ratio of progeny weaning weight to ewe breeding weight considered the fixed effect of year, and the random effect of ewe.

Least squares means for predicted ewe live weights every 50 days during their productive life were obtained with a linear model that included the fixed effects of day, age of dam, ewe birth rank, interaction between age of dam and ewe birth rank, and the interaction of day, age of dam and ewe birth rank.

Least squares means for predicted ewe live weight at breeding were obtained with a linear model that included the fixed effects of age of dam, ewe birth rank and year, and the interaction of age of dam and ewe birth rank.

Body condition score at breeding, number of lambs born per year, number of lambs weaned per year, and total lifetime number of lambs weaned were analysed using a generalised linear model, assuming a Poisson distribution and logit transformation. The model included the fixed effects of age of dam, ewe birth rank, and their interaction. Body condition score at breeding, number of lambs born per year, and number of lambs weaned per year also included the fixed effect of year, and the random effect of animal to account for repeated measures.

Lamb survival was analysed using a generalised linear model, assuming a binomial distribution and logit transformation. It included the fixed effects of age of dam, ewe birth rank, year, sex of lamb, and lamb birth rank, and the interaction of age of dam and ewe birth rank.

Survival analysis was carried out using exit data of all ewes that died or were removed prior to the end of the experiment (Day 2623). All ewes alive at the weaning of their 6th lamb (Day 2623) were censored at that date for the survival analysis. In addition, hypothetical culling was imposed, retrospectively, if a particular ewe was barren at pregnancy detection, day 40 of lactation, or weaning, as per commercial farming conditions. All ewes that were alive, and not culled at the end of the experiment were censored on day 2623 for the survival analysis.

## Results

### Measurements on the ewes

There was no significant interaction (P>0.05) between age of their dam and their birth rank for birth weight, crown rump length, or thoracic girth (Table 1). The ewes that were born to mature ewe dams were heavier (P<0.0001), and had greater (P<0.0001) crown rump length and thoracic girth than the ewes born to ewe lambs. The ewes that were born as singletons were heavier (P<0.0001), and had greater (P<0.0001) crown rump length and thoracic girth than the ewes born as twins.

**Fig 1:**
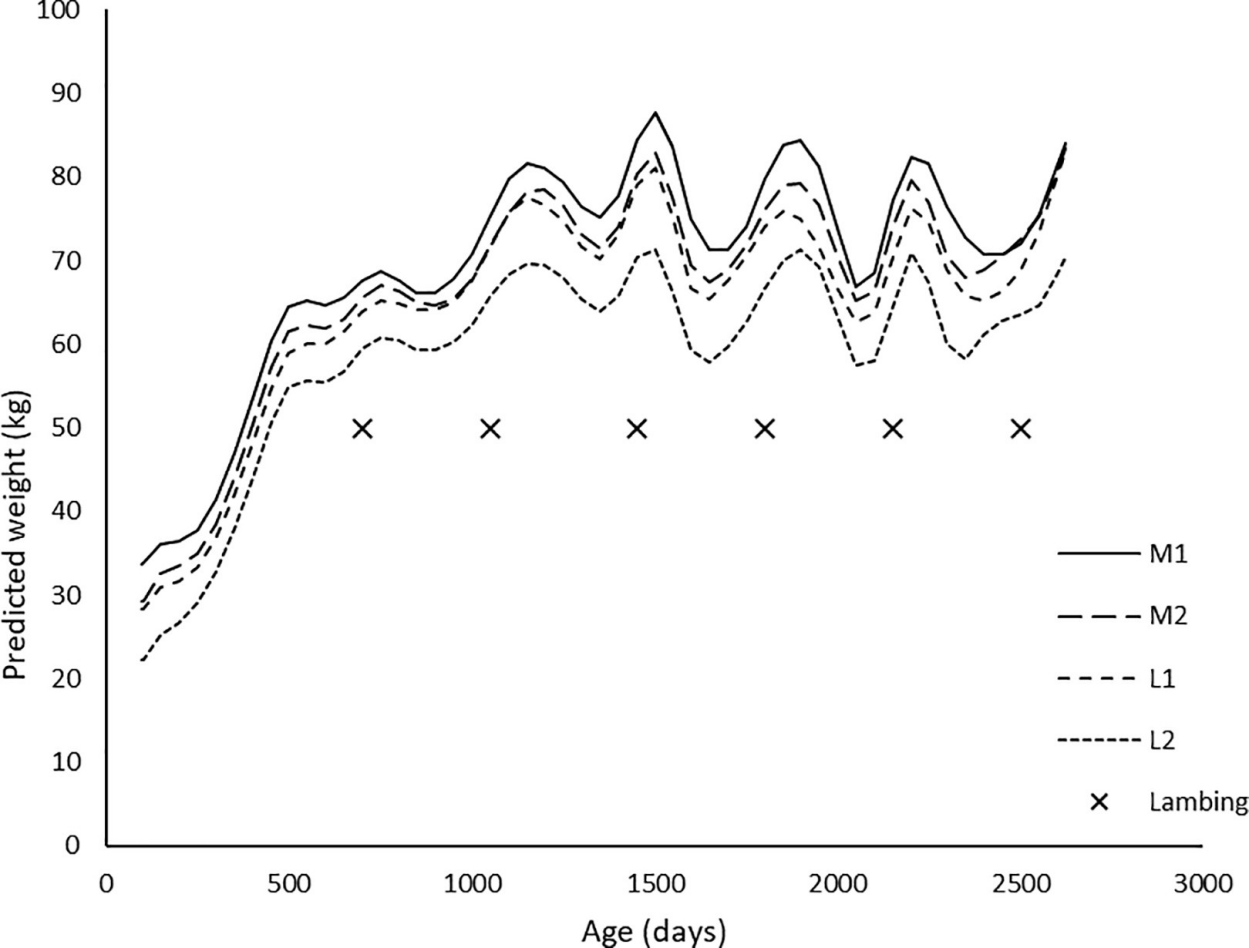
Spline fit (knots at breeding, lambing and weaning each year) predictions of daily live weight of ewes from their weaning (D99) to the weaning of their last lambs (D2623) for the interaction of dam age group and birth rank. M1 = singleton born to a mature ewe, M2 = twin born to a mature ewe, L1 = singleton born to a ewe lamb, and L2 = twin born to a ewe lamb. Times marked with an x indicate lambing dates each year (Days 692, 1057, 1442, 1790, 2146, and 2507).

The M1 ewes were consistently heaviest (P<0.05) throughout the experimental period, while M2 and L1 ewes were not significantly different (P>0.05) from each other, but were heavier (P<0.05) than L2 ewes (Fig 1).

**Table 1:** Least-squares means (± S.E.M) for weight, crown rump length, and thoracic girth at birth, and weight at Day 41 of life and weaning of ewes born as singles or twins to mature ewes or ewe lambs.

		n^4^	Birth weight (kg)	Crown-Rump length (cm)	Thoracic Girth (cm)	Day 41 of life weight (kg)	Weaning weight (kg)
Age of dam^1^							
	M	58	4.60 ± 0.12^b^	51.9 ± 0.5^b^	41.2 ± 0.4^b^	18.5 ± 0.4^b^	33.0 ± 0.6^b^
	L	57	3.77 ± 0.10^a^	47.6 ± 0.5^a^	37.6 ± 0.4^a^	14.4 ± 0.3^a^	27.0 ± 0.5^a^
	*P Value*		*<0*.*0001*	*<0*.*0001*	*<0*.*0001*	*<0*.*0001*	*<0*.*0001*
Birth rank^2^							
	1	45	4.55 ± 0.12^b^	51.9 ± 0.6^b^	40.6 ± 0.4^b^	18.3 ± 0.4^b^	32.7 ± 0.6^b^
	2	70	3.82 ± 0.10^a^	47.6 ± 0.4^a^	38.1 ± 0.3^a^	14.5 ± 0.3^a^	27.2 ± 0.5^a^
	*P Value*		*<0*.*0001*	*<0*.*0001*	*<0*.*0001*	*<0*.*0001*	*<0*.*0001*
Interaction^3^							
	M1	17	4.94 ± 0.20^c^	54.2 ± 0.9^c^	42.6 ± 0.7^c^	20.3 ± 0.7^c^	35.2 ± 1.0^c^
	M2	41	4.27 ± 0.12^b^	49.6 ± 0.6^b^	39.7 ± 0.4^b^	16.6 ± 0.4^b^	30.8 ± 0.6^b^
	L1	28	4.16 ± 0.15^b^	49.7 ± 0.7^b^	38.6 ± 0.5^b^	16.3 ± 0.5^b^	30.3 ± 0.8^b^
	L2	29	3.37 ± 0.15^a^	45.5 ± 0.7^a^	36.5 ± 0.5^a^	12.5 ± 0.5^a^	23.6 ± 0.7^a^
	*P Value*		*0*.*6947*	*0*.*7896*	*0*.*4525*	*0*.*9423*	*0*.*1617*

^1^Dam age group: M = mature ewe, L = ewe lamb

^2^dam birth rank: 1 = singleton, 2 = twin

^3^interaction of dam age group and dam birth rank: M1 = singleton born to a mature ewe, M2 = twin born to a mature ewe, L1 = singleton born to a ewe lamb, and L2 = twin born to a ewe lamb

^4^number of ewes.

Values within columns with different superscripts (^a,b,c^) are significantly different (P<0.05).

The interaction of dam age group and birth rank was not significant (P>0.05) for live weight or body condition score at joining (Table 2). Ewes born to mature ewe dams were heavier (P<0.0001) and had greater (P<0.0001) body condition score at breeding than ewes born to ewe lambs. Ewes born as singletons were heavier (P<0.002) at breeding than ewes born as twins. There was no difference (P>0.05) in body condition score for ewes of different birth ranks.

**Table 2:** Least-squares means (± S.E.M.) for ewe live weight and body condition score at breeding over their lifetime based on age of their dam, and their birth rank, and the interaction of dam age group and ewe birth rank.

		n^4^	Live weight (kg)	Body condition score
Age of dam^1^				
	M	348	69.0 ± 0.5^b^	2.91 (2.80–3.02)^b^
	L	342	62.7 ± 0.5^a^	2.69 (2.61–2.77)^a^
	*P Value*		*<0*.*0001*	*0*.*0014*
Birth rank^2^				
	1	270	68.0 ± 0.5^b^	2.83 (2.73–2.93)
	2	420	63.8 ± 0.4^a^	2.77 (2.68–2.86)
	*P Value*		*<0*.*0001*	*0*.*3087*
Interaction^3^				
	M1	102	70.6 ± 0.8^d^	2.98 (2.83–3.13)^b^
	M2	246	67.5 ± 0.5^c^	2.84 (2.70–2.99)^ab^
	L1	168	65.3 ± 0.7^b^	2.69 (2.58–2.82) ^a^
	L2	174	60.1 ± 0.7^a^	2.69 (2.60–2.79)^a^
	*P Value*		*0*.*1155*	*0*.*3177*

^1^Dam age group: M = mature ewe, L = ewe lamb

^2^dam birth rank: 1 = singleton, 2 = twin

^3^interaction of dam age group and dam birth rank: M1 = singleton born to a mature ewe, M2 = twin born to a mature ewe, L1 = singleton born to an ewe lamb, and L2 = twin born to an ewe lamb

^4^number of ewe records.

Values within columns with different superscripts (^a,b,c,d^) are significantly different (P<0.05).

### Lamb production

Number of lambs born, number of lambs weaned, and lamb survival did not differ (P>0.05) between ewe groups (Table 3). There was no significant interaction (P>0.05) of dam age group and ewe birth rank on the total litter weight at birth and day 40 of lactation (Table 4). Ewes born to mature ewes had lighter (P<0.05) litters at birth, but litter weights did not differ (P>0.05) at 40 days of age. There was a significant interaction of dam age group and ewe birth rank on the total litter weight at weaning (P<0.02). Ewes that were born to mature ewes as either singletons or twins were not different (P>0.05). Twin-born ewes born to ewe lambs had lighter litters than single-born ewes.

**Table 3:** Least-squares means (95% confidence interval) for the mean number of lambs born (NLB) and weaned (NLW) per year, and lamb survival (%) for dam age group, ewe birth rank, and the interaction of dam age group and ewe birth rank.

		n^4^	NLB /ewe	NLW/ewe	Lamb survival (%)
Dam age group^1^					
	M	348	1.51 (1.42–1.62)	1.48 (1.38–1.58)	85.2 (80.5–88.8)
	L	342	1.51 (1.42–1.61)	1.42 (1.33–1.53)	80.9 (75.6–85.2)
	*P Value*		*0*.*9880*	*0*.*4513*	*0*.*1342*
Birth rank^2^					
	1	270	1.49 (1.39–1.59)	1.47 (1.37–1.58)	84.4 (79.3–88.4)
	2	420	1.54 (1.46–1.63)	1.43 (1.34–1.53)	81.8 (77.1–85.7)
	*P Value*		*0*.*4166*	*0*.*5528*	*0*.*3679*
Interaction^3^					
	M1	102	1.47 (1.32–1.63)	1.46 (1.30–1.64)	84.9 (77.0–90.5)
	M2	246	1.51 (1.38–1.65)	1.48 (1.36–1.62)	85.4 (80.4–89.2)
	L1	168	1.56 (1.45–1.69)	1.49 (1.40–1.59)	83.8 (77.4–88.6)
	L2	174	1.52 (1.40–1.65)	1.37 (1.23–1.53)	77.6 (69.8–83.8)
	*P Value*		*0*.*5232*	*0*.*2910*	*0*.*2942*

^1^Dam age group: M = mature ewe, L = ewe lamb

^2^dam birth rank: 1 = singleton, 2 = twin

^3^interaction of dam age group and dam birth rank: M1 = singleton born to a mature ewe, M2 = twin born to a mature ewe, L1 = singleton born to a ewe lamb, and L2 = twin born to a ewe lamb

^4^number of ewe records.

**Table 4:** The effect of dam age group and ewe birth rank on total litter weight (kg) per year at birth, day 40 of lactation, and weaning, and ratio of progeny weaning weight to ewe breeding weight. Data presented are least squares means (± S.E.M).

		n^4^	Litter birth weight (kg)	Litter day 40 of lactation weight (kg)	Litter weaning weight (kg)	Ratio of progeny weaning weight to ewe breeding weight
Dam age group^1^						
	M	314	8.51 ± 0.15^a^	21.0 ± 0.6	48.8 ± 1.2	0.713 ± 0.019
	L	286	8.92 ± 0.15^b^	19.9 ± 0.6	46.3 ± 1.2	0.745 ± 0.019
	*P Value*		*0*.*0476*	*0*.*1672*	*0*.*1434*	*0*.*2235*
Birth rank^2^						
	1	241	8.58 ± 0.16	20.4 ± 0.6	47.7 ± 1.3	0.709 ± 0.021
	2	359	8.85 ± 0.13	20.5 ± 0.5	47.4 ± 1.1	0.749 ± 0.017
	*P Value*		*0*.*2001*	*0*.*9303*	*0*.*8241*	*0*.*1396*
Interaction^3^						
	M1	96	8.29 ± 0.25^a^	20.8 ± 0.9	47.5 ± 2.0^ab^	0.675 ± 0.032
	M2	218	8.73 ± 0.16^ab^	21.2 ± 0.6	50.1 ± 1.4^b^	0.750 ± 0.021
	L1	145	8.87 ± 0.20^ab^	20.1 ± 0.8	48.0 ± 1.7^ab^	0.743 ± 0.026
	L2	141	8.97 ± 0.20^b^	19.8 ± 0.8	44.6 ± 1.7^a^	0.747 ± 0.027
	*P Value*		*0*.*4063*	*0*.*6230*	*0*.*0745*	*0*.*1786*

^1^Dam age group: M = mature ewe, L = ewe lamb

^2^dam birth rank: 1 = singleton, 2 = twin

^3^interaction of dam age group and dam birth rank: M1 = singleton born to a mature ewe, M2 = twin born to a mature ewe, L1 = singleton born to a ewe lamb, and L2 = twin born to a ewe lamb

^4^n number of ewe records.

Values within columns with different superscripts (^a,b^) are significantly different (P<0.05).

### Ewe efficiency

There was no significant interaction (P>0.05) between dam age group and ewe birth rank on estimated total volume of pasture eaten, weight of lamb weaned, and efficiency of conversion of pasture to lamb growth over the eight years (Table 5). Calculated feed intake of ewes born to mature ewes was greater (P<0.05) in their lifetime than ewes that were born to ewe lambs. Calculated feed intake of ewes born as singletons was greater (P<0.05) in their lifetime than ewes that were born as twins. There was no effect (P>0.05) of dam age group or ewe birth rank on lifetime total progeny weaning weight or the efficiency of lamb production for estimated lifetime ewe intake. The total number of lambs weaned was similar (P>0.05) between all ewe groups.

**Table 5:** The effect of dam age group and ewe birth rank on the lifetime total predicted pasture consumption (kgDM), total lifetime progeny weaning weight (kg), total lifetime number of lambs weaned and efficiency of lamb production (total lamb weaning weight divided by predicted pasture eaten). Data presented are least squares means (± S.E.M) for pasture, weaning weight and efficiency, and least squares means (95% confidence intervals) for number of lambs weaned.

		n^4^	Total lifetime predicted pasture eaten (kgDM)	Total lifetime weaning weight (kg)	Total number of lambs weaned	Efficiency (%)
Dam age group^1^						
	M	58	4620 ± 180^b^	232 ± 14	7.12 (6.41–7.92)	4.88 ± 0.21
	L	57	4000 ± 170^a^	203 ± 13	6.26 (5.64–6.94)	4.64 ± 0.19
	*P Value*		*0*.*0140*	*0*.*1257*	*0*.*0859*	*0*.*3992*
Birth rank^2^						
	1	45	4490 ± 190	222 ± 15	6.94 (6.19–7.78)	4.75 ± 0.22
	2	70	4140 ± 150	213 ± 12	6.42 (5.84–7.06)	4.76 ± 0.17
	*P Value*		*0*.*1677*	*0*.*6273*	*0*.*3032*	*0*.*9738*
Interaction^3^						
	M1	17	4690 ± 310^b^	230 ± 24	7.18 (6.01–8.57)^ab^	4.80 ± 0.35
	M2	41	4560 ± 200^b^	235 ± 15	7.07 (6.30–7.94)^b^	4.96 ± 0.22
	L1	28	4280 ± 240^ab^	215 ± 19	6.71 (5.82–7.75)^ab^	4.71 ± 0.27
	L2	29	3720 ± 240^a^	191 ± 18	5.83 (5.01–6.78)^a^	4.57 ± 0.27
	*P Value*		*0*.*3925*	*0*.*4325*	*0*.*3995*	*0*.*6022*

^1^Dam age group: M = mature ewe, L = ewe lamb

^2^dam birth rank: 1 = singleton, 2 = twin

^3^interaction of dam age group and dam birth rank: M1 = singleton born to a mature ewe, M2 = twin born to a mature ewe, L1 = singleton born to a ewe lamb, and L2 = twin born to a ewe lamb

^4^number of ewes.

Values within columns with different superscripts (^a,b^) are significantly different (P<0.05).

### Ewe longevity

**Fig 2:**
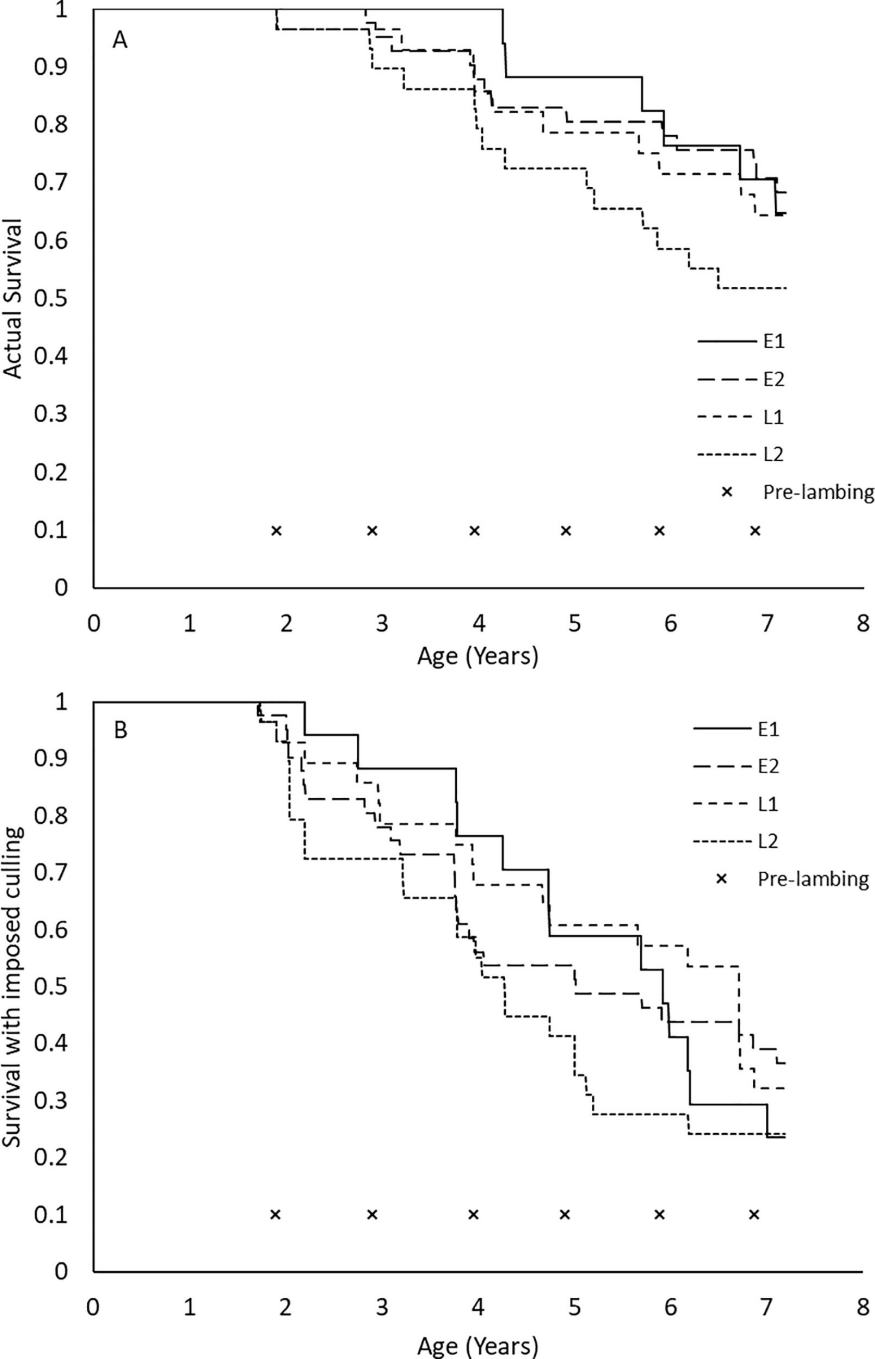
Survival curves of the ewes based on the interaction of dam age group (mature ewe or ewe lambs) and birth ranks (singleton or twin) for the eight years of the experiment, with actual survival, and imposed survival. **(A)** The actual survival of the ewes, with no culling, except for welfare grounds. **(B)** Imposed culling, with culling for production traits, as per commercial farm conditions. M1 = singleton born to a mature ewe, M2 = twin born to a mature ewe, L1 = singleton born to a ewe lamb, and L2 = twin born to a ewe lamb. Times marked with an x indicate lambing dates each year (Days 692, 1057, 1442, 1790, 2146, and 2507).

When no culling occurred, the L2 ewes had the lowest (P<0.05) proportion of survival of all the ewes (Fig 2A). The M1 ewes had no deaths until their third set of lambs, at 4 years of age. However, with culling retrospectively imposed, survival proportions were much lower (P<0.05) than the actual survival of the ewes (Fig 2B), for all dam ages and birth ranks. If culling was imposed, M1 ewes were initially culled at weaning of their first lambs, as two-year olds, rather than as four-year-olds, after the weaning of their third lambs.

## Discussion

The aims of this experiment were to determine the effects of dam age and birth rank on the lifetime performance of female offspring and to extend the data of Loureiro [17] which only followed these ewes to 2.5 years of age. Ewes that were born as twins to ewe lambs were lighter at birth, and throughout their lifetime compared to ewes born as singles to ewe lambs or as singletons or twins born to mature ewes. This supports previous studies that show lambs born as twins are smaller at birth than singles [24, 25], and lambs born to ewe lambs are smaller than lambs that are born to mature ewes [24, 26–28]. However, it adds that there is an interaction of age of dam and birth rank on the ewe’s live weights, with twins born to ewe lambs having additive effects on their live weight. Lambs born to ewe lambs are lighter than lambs born to mature ewes, due to the maternal constraint of body size of the ewe lambs. This is a result of the maturity of the ewe lamb, which is still growing to mature size while pregnant, and has different nutrient partitioning than a mature ewe, who is no longer growing [29]. Combined with the effect of being born as a twin, the L2 ewes are smaller at birth than the M2 ewes, and from the results of this study, these effects appear to persist for their lifetime. Body condition scores [19] did not differ during the experimental period, indicating that the lighter body weights are not due to poorer condition. This suggests that the 10 kg difference in live weight might be explained by differences in their mature frame, and this warrants investigation in future studies.

Previous studies have shown that ewe reproductive traits, such as ovulation rate [30], reproductive rate (foetuses/100 ewes bred) [31], and lamb birth and weaning weights [32] are affected by live weight of the ewe, with heavier ewes having more lambs, which are heavier at birth and weaning. Therefore, it was expected that the L2 ewes would be disadvantaged in their reproductive performance, because they were 10 kg lighter than the M1 ewes. However, there was no difference in reproductive rates between the L2 and M1 ewes. The L2 ewes had heavier litter birth weights, but lighter litter weaning weights than the M1 ewes. This is possibly due to the lower rates of lamb survival between the L2 ewes and the other three groups. The L2 ewes had a similar number of lambs born, but weaned 0.1 less lambs than the other three groups, leading to lighter litter weaning weights than the other ewe groups.

Even though the L2 ewes were 10 kg lighter than the M1 ewes at breeding, this was not enough to significantly increase the ratio of litter weaning weight to ewe weight at breeding. The L2 ewes also had a lower predicted pasture consumption, but weaned fewer lambs for their lifetime, resulting in a similar efficiency to the M1 ewes. While the L2 ewes were not more efficient, there was no loss of production from having lighter live weights. However, because of the small group sizes further investigation, with larger group sizes, is warranted, to increase the statistical power, especially with binomial traits. Given the results in this study, a sample size of 189 per group would be required to show a statistically significant difference in efficiency between the groups.

The lighter live weights of L2 ewes with similarity in production indicates farmers could have more of these types of ewes on their farm for a given total feed availability, and subsequently produce greater weight of lamb per hectare. However, a more powerful study, with an economic evaluation would be warranted to determine the economic benefits for farmers. In New Zealand, the Sheep Improvement Limited (SIL) dual purpose index indicates ewe live weight for feed to have a value of $-0.14/kg, indicating lighter ewes are more economical to feed [33]. Additionally, selecting replacements that are born to ewe lambs can increase the rate of genetic gain, with a shorter generation interval (younger parents) and greater selection intensity (more animals to select from) [34]. While it is not practical to select all lambs from ewe lambs, this result gives some incentive for farmers to breed their ewe lambs, and select some of their replacements from the lambs produced, especially those that are born as singles.

Ewe survival tended to be lowest in L2 ewes compared to the other ewe groups, with losses occurring earlier in these ewes than in the other groups. This may have decreased their lifetime number and weaning weight of lambs produced, but it will also decrease their lifetime estimated pasture consumption. With sharp decreases in ewe numbers associated with lambing time, there are many ewes being fed from the weaning of their previous lamb until lambing, without any lambs being weaned, which may decrease efficiency of the L2 ewes.

Previous studies [18, 35] have shown that the average longevity of ewes under commercial conditions is 4.3–5.0 years of age, with the average rate of loss within flock being between 4.6–4.9% per year [36]. The M1 ewes had lower rates of reproductive performance than the other ewe groups, which is more apparent when looking at the survival curve when culling was imposed. This indicates that these ewes are producing fewer lambs, with high rates of barrenness. Ewe lambs that were detected to be showing oestrous prior to breeding, but were not bred, were heavier at their next breeding than ewe lambs that showed oestrous, were bred, and did rear a lamb [37]. Therefore barrenness in the M1 ewes may be a cause for their larger live weights throughout their lifetime, as they were not disadvantaged by pregnancy and lactation.

## Conclusions

Ewes that are born to ewe lambs as twins are lighter than ewes born to ewe lambs as singles and ewes that are born to mature ewes as twins, which are lighter than ewes born to mature ewes as singles, for their lifetime to six-and-a-half years of age. The L2 ewes have the fastest rate of mortality, and M1 ewes have the slowest rate of mortality. The live weights of L2 ewes may not affect litter weight at weaning, producing similar weights of lambs weaned as the M1 ewes. However, this is insufficient to increase the efficiency of the L2 ewes compared to the other ewe groups, but consequently may not impair their production. Therefore, farmers could reasonably select lambs born to ewe lambs as replacements for their flock, without compromising their production. Further investigation is warranted, with a larger dataset and greater statistical power, to confirm the effects of selecting lambs born to ewe lambs, on their production and survival. An economic analysis is now required to determine whether there is sufficient financial benefit to farmers to warrant them implementing a policy of mating ewe lambs.

## Supporting information

S1 Appendix The equations used to determine daily energy requirements for ewes, including maintenance requirements, liveweight gain or loss, requirements for gestation, and requirements for lactation, based on daily live weights of the ewes, and live weights and average daily gains of the lambs. (DOCX)
