# Supplementary material for: The effects of birth rank (single or twin) and dam age on the lifetime productive performance of female dual purpose sheep (Ovis aries) offspring in New Zealand
Source: PLoS One. 2019 Mar 21;14(3):e0214021. doi: 10.1371/journal.pone.0214021 (PMC6428402; doi:10.1371/journal.pone.0214021)
Supplement: S1 Appendix — (DOCX) [file pone.0214021.s001.docx]

# S1 Appendix – Energy requirement equations

## Maintenance

$\mathrm{Maintenance}=0.52 x Predicted liveweight^0.75$

## Liveweight gain or loss

$Liveweight gain=55 x change in liveweight$

$Liveweight loss=30 x change in liveweight$

## Gestation requirement

$y=\frac{Total birth weight}{4} x Exp(7.64-11.46 x \left( \mathrm{Exp}\left( -0.00643 x Day of gestation \right) \right))$

$\frac{\mathrm{dt}}{\mathrm{dy}}=11.46 x 0.00643 x Exp\left( -0.00643 x Day of gestation \right) x y$

$Conversion of energy to gestational growth=0.133$

$Daily gestational requirement=\frac{\frac{\mathrm{dy}}{\mathrm{dt}}}{0.133}$

## Lactation requirement

Daily milk yield was predicted from [21] for days in milk, and number of lambs reared.

Lamb growth was assumed linear from birth to weaning.

$$Lamb average daily gain \left( \mathrm{ADG} \right)=\frac{(Weaning weight-birth weight)}{Age at weaning}$$

$Energy in milk=\left( 0.0328 x 0.08+0.0025 x day of lactation+2.203 \right) x Milk yield$

$Conversion of energy to milk production=0.6$

$Conversion of energy in milk to lamb growth=0.38$

$Energy consumed by the ewe to make milk=\frac{Energy in milk}{0.6}$

$Energy consumed by the lamb from milk=Energy in milk x 0.38$

$$Additional lamb requirement from grass=\left( Lamb maintenance+Lamb growth \right)-Energy from milk$$

## Total daily requirement

$$Total energy=Ewe mainenance+Ewe liveweight change+Energy to make milk+additional lamb requirements$$

## Other assumptions

- Lambs that did not have a birth weight (n=2), and/or date of birth (n=1), were given a birth weight average and/or date of birth average, respectively, for the ewe group for the year they were born.
- Lambs that did not have a weaning weight were assumed to have died. Any lambs that died were assumed to have died at birth, and had no energy requirements from birth to weaning.
- Ewes that died prior to pregnancy detection were assumed to be non-pregnant.
- Ewes that died between pregnancy detection and lambing, and were carrying one or more lambs, were given an average lamb birth weight and date of birth for their ewe group of the year they died to calculate gestational energy requirements prior to death.
